# Supplementary material for: Cancer cell-mitochondria hybrid membrane coated Gboxin loaded nanomedicines for glioblastoma treatment
Source: Nat Commun. 2023 Jul 28;14:4557. doi: 10.1038/s41467-023-40280-3 (PMC10382535; doi:10.1038/s41467-023-40280-3)
Supplement: Supplementary file 1 — Supplementary Information [file 41467_2023_40280_MOESM1_ESM.pdf]

Cancer cell-mitochondria hybrid membrane coated Gboxin loaded nanomedicines for glioblastoma treatment

Yan Zou[1,2], Yajing Sun[1], Yibin Wang[1], Dongya Zhang[1], Huiqing Yang[1], Xin Wang[1], Meng Zheng[1], Bingyang Shi[1,2*]

[1]Henan-Macquarie University Joint Centre for Biomedical Innovation, Henan Key Laboratory of Brain Targeted Bio-nanomedicine, School of Life Sciences, Henan University, Kaifeng, Henan, 475004, China

[2]Centre for Motor Neuron Disease Research, Macquarie Medical School, Faculty of Medicine, Human Health Sciences, Macquarie University, Sydney, NSW 2109, Australia

*Corresponding author. bingyang.shi@mq.edu.au (B. Shi).

Supplementary Table 1. Characterization of nanoparticles with different theoretical drug loading content

Theoretical drug loading content (%)	DLE[a] (%)	DLC[a] (%)	Size[b] (nm)	PDI[b]	Zeta[c] (mV)
10	41.5	4.4	63.4 ±0.5	0.21 ±0.01	2.9 ±0.2
20	70.4	15.0	67.3 ±2.1	0.19 ±0.07	4.1 ±0.9
30	44.9	16.1	72.5 ±1.4	0.19 ±0.02	8.3 ±0.2

[a]Determined by HPLC, mobile phase: A: ddH_2O (0.01% TFA), B: acetonitrile (0.01% TFA), gradient: 5% to 95% B within 1.3 min, 95% to 5% B from 1.3 min to 3.0 min, flow rate: 1.8 mL min^{-1}, injection volume: 10 µL, Colum: SunFire C18 (50 mm × 4.6 mm, 3.5 µm), oven temperature: 45 °C.

[b]Determined by DLS (10 mW He-Ne laser, 633 nm wavelength), [c] determined using Zetasizer Nano-ZS (Malvern instruments) at 25 °C in HEPES buffer (pH 7.4, 10 mM).

Supplementary Table 2. Characterization of membrane coated nanoparticles

Entry	Size[a] (nm)	PDI[a]	Zeta[b] (mV)
HM-NPs@G	89.1 ±1.5	0.22 ±0.01	-25.6 ±0.3
MM-NPs@G	88.2 ±0.7	0.17 ±0.01	-24.3 ±0.2
CM-NPs@G	93.3 ±1.9	0.19 ±0.02	-24.9 ±0.6

Theoretical drug loading content (DLC) of Gboxin is 20 wt.%.

[a]Determined by DLS (10 mW He-Ne laser, 633 nm wavelength), [b] determined using Zetasizer Nano-ZS (Malvern instruments) at 25 ℃ in HEPES buffer (pH 7.4, 10 mM).

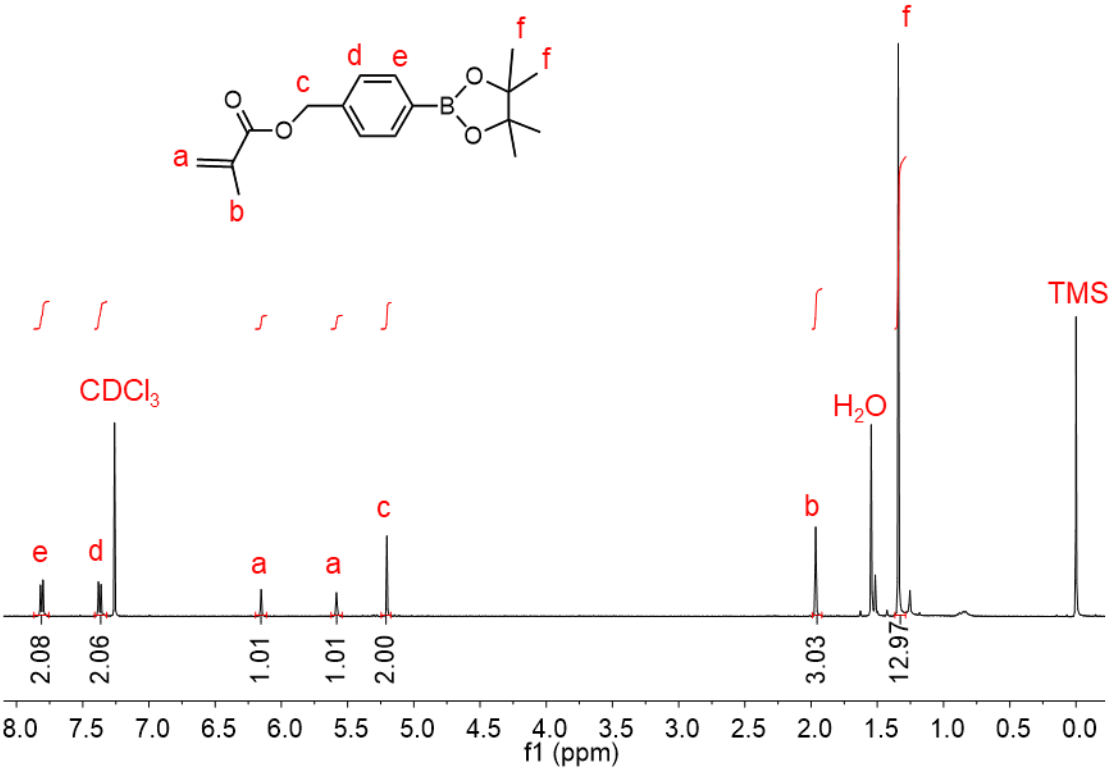

Supplementary Figure 1 ^{1}H NMR analysis of HB monomer. ^{1}H NMR spectrum of HB monomer ($CDCl_3$, 400 MHz).

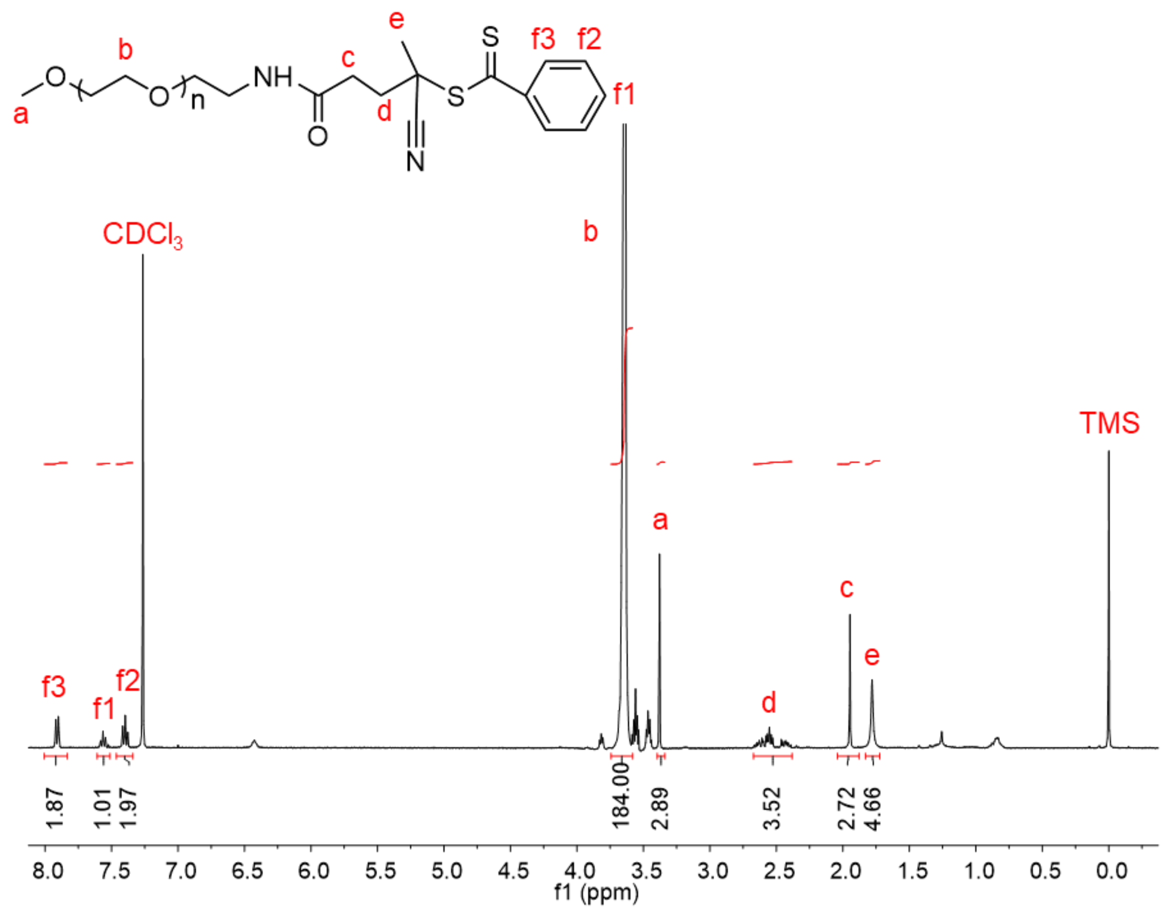

Supplementary Figure 2 ^{1}H NMR analysis of PEG-RAFT. ^{1}H NMR spectrum of PEG_{2k}-RAFT ($CDCl_3$, 400 MHz).

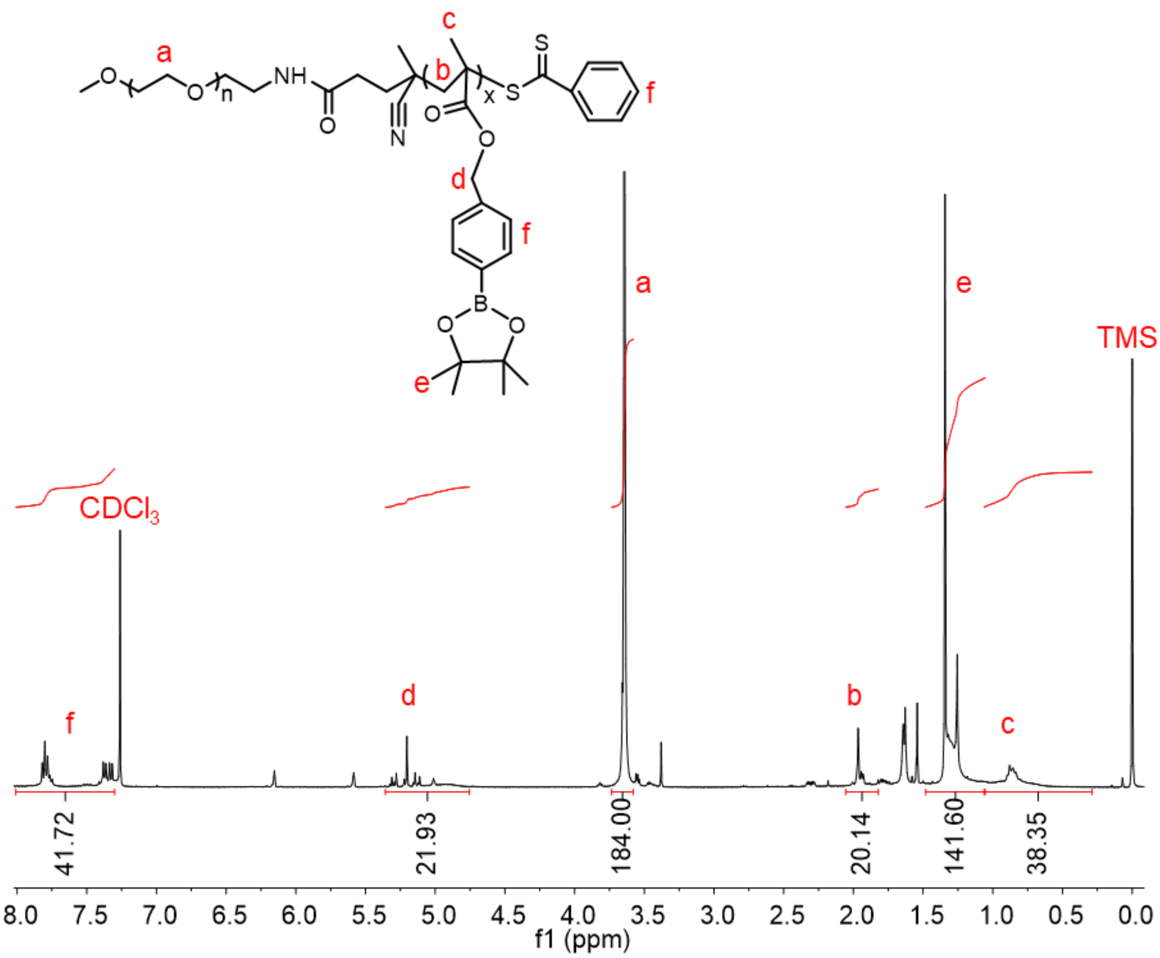

Supplementary Figure 3 ^{1}H NMR analysis of PEG-PHB. ^{1}H NMR spectrum of PEG-PHB block polymer ($CDCl_3$, 400 MHz).

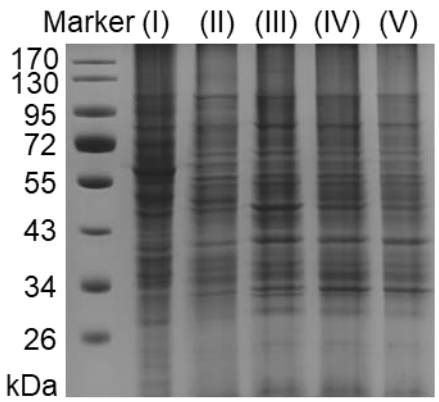

Supplementary Figure 4 SDS-PAGE protein detection. SDS-PAGE protein analysis of U87MG cancer cell lysates (I), CM (II), MM (III), HM (IV) and HM from after HM-NPs@G treatment (V). Samples were run at equal protein concentration and stained with Coomassie Blue. SDS-PAGE protein analysis was representative data from three independent experiments.

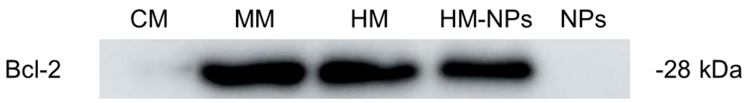

Supplementary Figure 5 Bcl-2 expression analysis. Western blot analysis showing the Bcl-2 expression on the cancer cell membrane (CM), mitochondria membrane (MM), cancer cell-mitochondria hybrid membrane (HM), hybrid membrane coated nanoparticles (HM-NPs), and NPs, respectively. The immunoblot was representative data from three independent experiments.

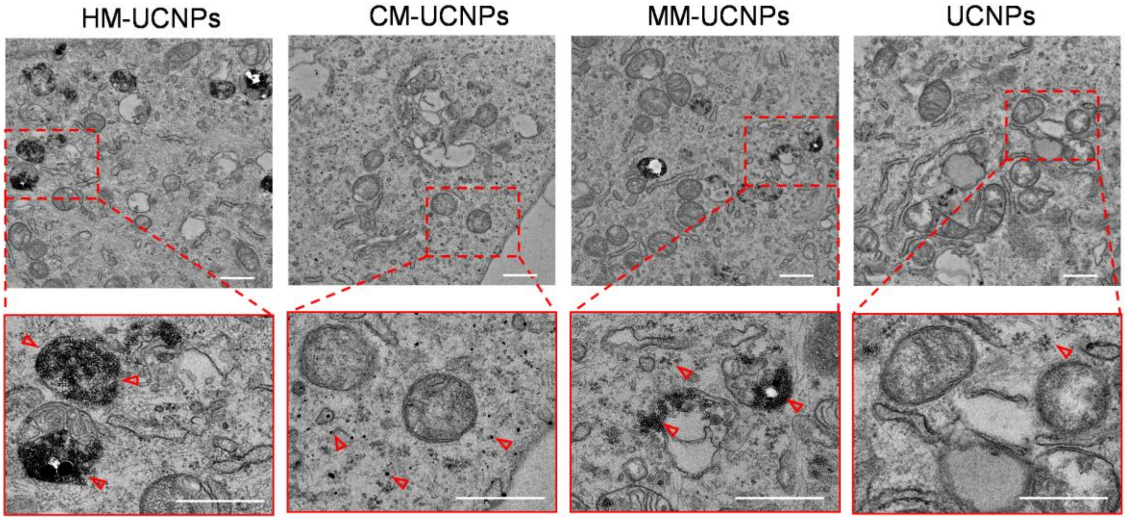

Supplementary Figure 6 Co-localization analysis by Bio-TEM. TEM images of mitochondria in U87MG cells treated with HM-UCNPs, CM-UCNPs, MM-UCNPs and bare UCNPs. UCNPs: 1 mg/mL. Scale bar = 500 nm. The red arrows indicated the UCNPs. The TEM images were representative data from three independent experiments.

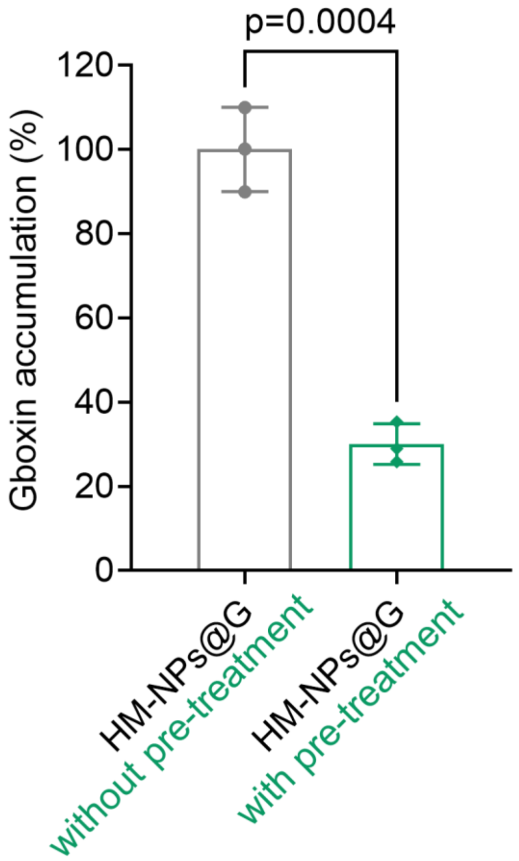

Supplementary Figure 7 Mitochondria targeting and penetrating analysis of HM-NPs@G in vitro. The content of Gboxin in mitochondria isolated from U87MG cells, with or without MFI8 pre-treatment, followed by incubated with HM-NPs@G for 24 h (Gboxin: 20 μg mL^{-1}, n = 3). Free MFI8 was pre-incubated with U87MG cells for 6 h (MFI8: 5 μM). The Gboxin accumulation of HM-NPs@G with pre-treatment is relative to the HM-NPs@G without pre-treatment. (n = 3 biologically independent samples). Data are presented as mean ± SD. (Statistical analysis was performed using two-sample t-test). Source data are provided as a Source Data file.

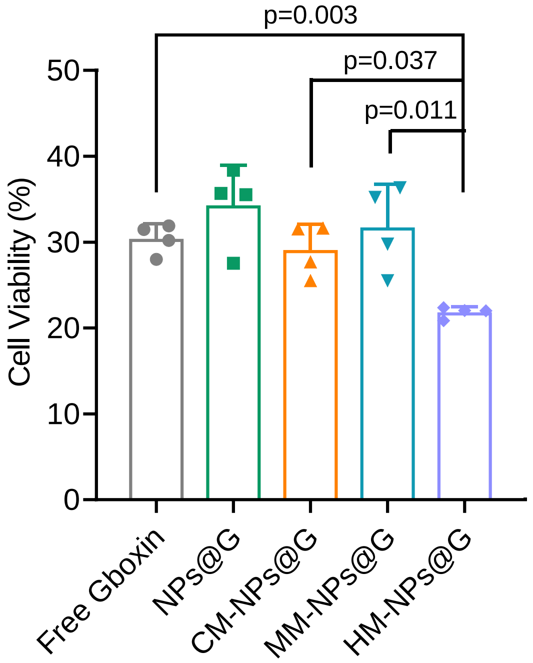

Supplementary Figure 8 Cell viability analysis of X01 cells treated with different treatments. Cell viability of X01 after 72 h incubation with HM-NPs@G, MM-NPs@G, CM-NPs@G and free Gboxin (Gboxin: 800 nM). Data were normalized to cells incubated with PBS (n=4). Data are presented as mean ± SD (n = 4 biologically independent samples; one-way ANOVA and Tukey's multiple comparison test). Source data are provided as a Source Data file.

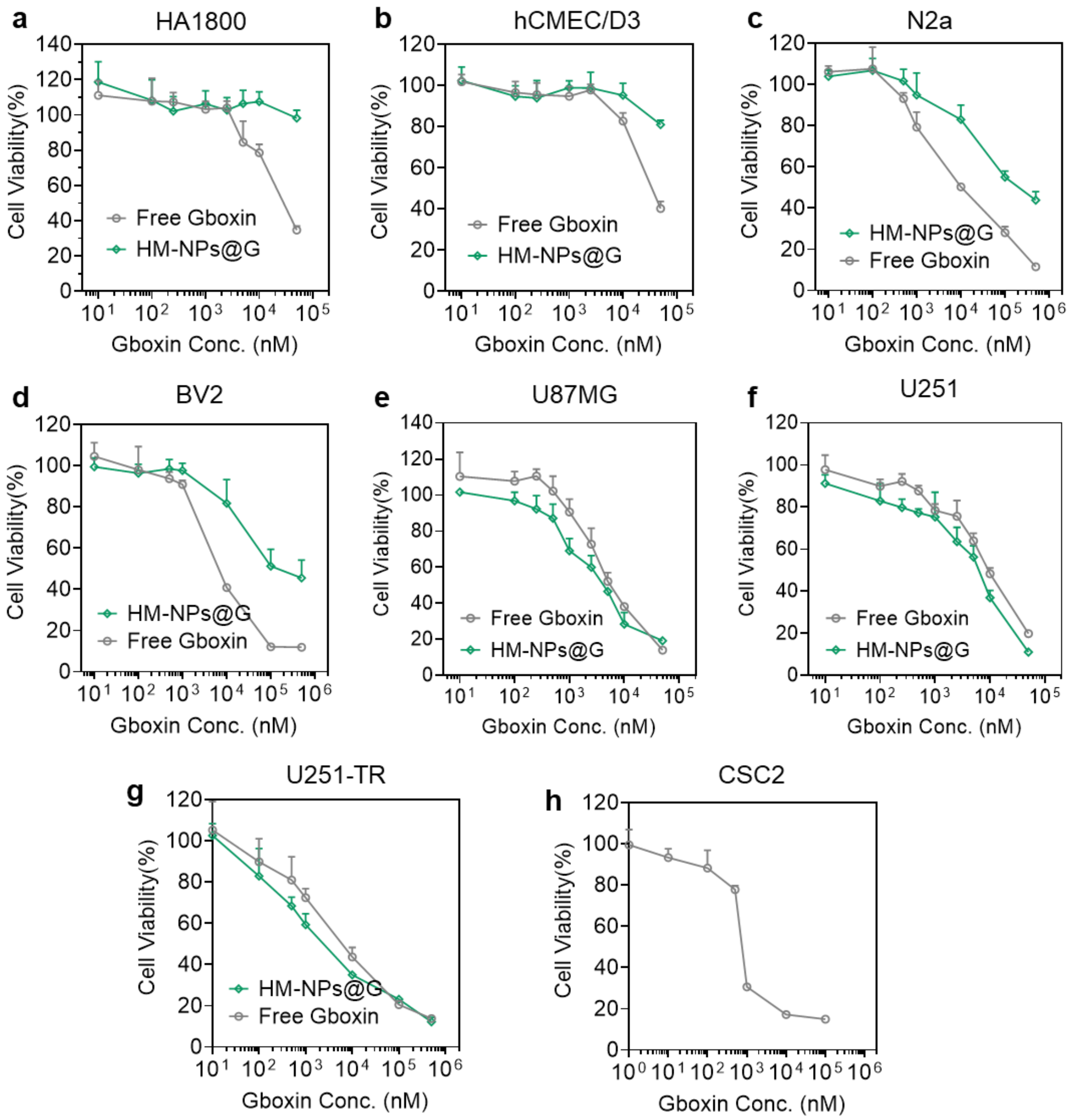

Supplementary Figure 9 IC_{50} assay in different cell lines. IC_{50} analysis of normal cells including **(a)** HA1800 (n = 5 biologically independent samples), **(b)** hCMEC/D3 (n = 5 biologically independent samples), **(c)** N2a (n = 5 biologically independent samples), and **(d)** BV2 cell lines (n = 4 biologically independent samples), glioblastoma tumor cells (**(e)** U87MG (n = 4 biologically independent samples), **(f)** U251 (n = 5 biologically independent samples) and **(g)** U251-TR cell lines (n = 5 biologically independent samples)) as well as **(h)** glioblastoma stem cells (X01) (n = 4 biologically independent samples) after 72 h incubation

with HM-NPs@G and free Gboxin. Data were normalized to cells incubated with PBS. Data are presented as mean ± SD. Source data are provided as a Source Data file.

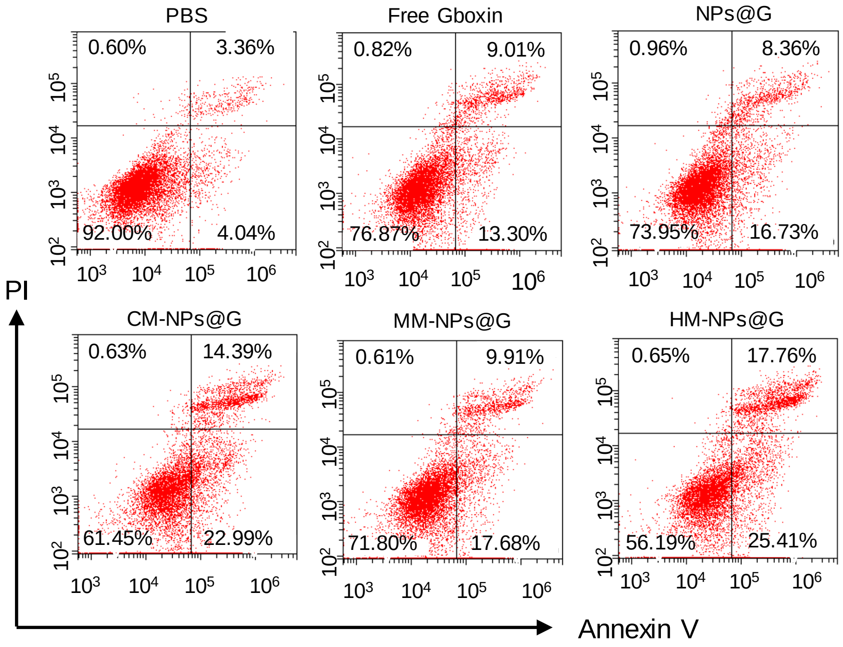

Supplementary Figure 10 Apoptosis analysis of U87MG cells. Apoptosis analysis of U87MG cells after 72 h incubation with free Gboxin, NPs@G, CM-NPs@G, MM-NPs@G, or HM-NPs@G (Gboxin: 800 nM). The apoptosis analyses were representative data from three independent experiments.

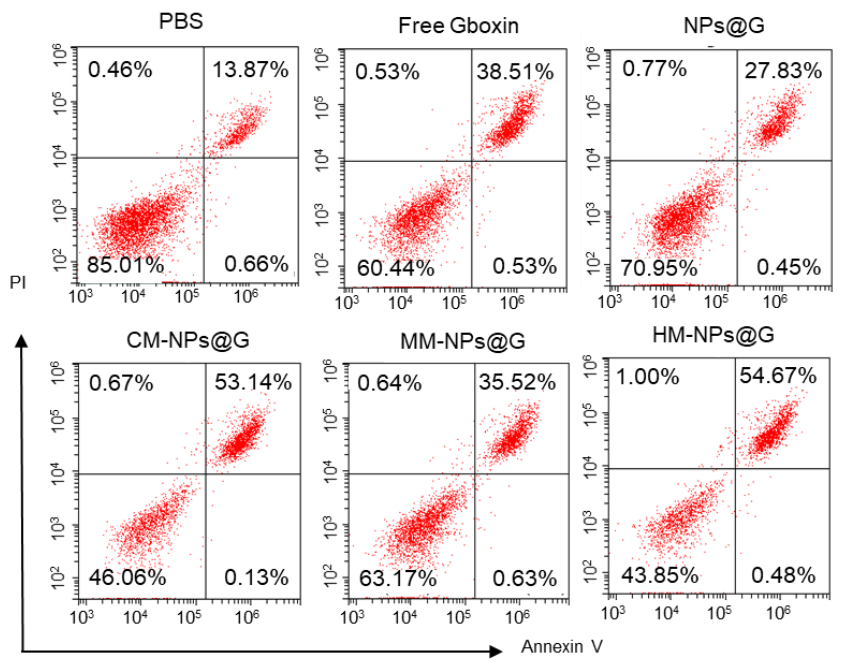

Supplementary Figure 11 Apoptosis analysis of X01 cells. Cell apoptosis of GBM stem cells (GSCs) X01 after 72 h incubation with HM-NPs@G, MM-NPs@G, CM-NPs@G and free Gboxin (Gboxin: 800 nM). The apoptosis analyses were representative data from three independent experiments.

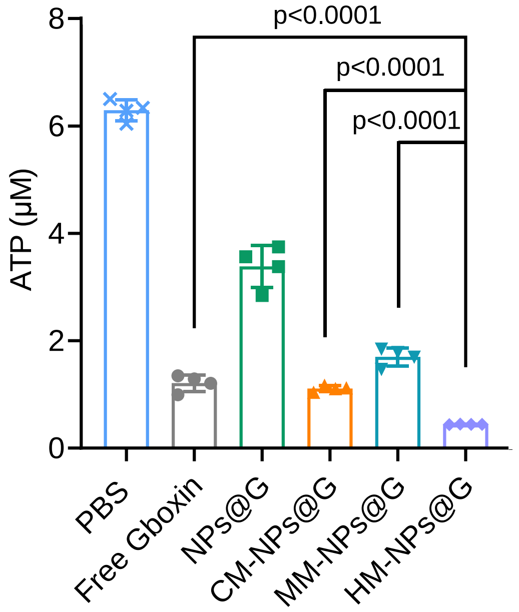

Supplementary Figure 12 ATP level detection of X01 cells treated with different treatments. ATP level of X01 cells when treated with HM-NPs@G, MM-NPs@G, CM-NPs@G, free Gboxin (Gboxin: 800 nM) and PBS for 72 h (n=4 biologically independent samples). Data are presented as mean ± SD (one-way ANOVA and Tukey multiple comparisons tests). Source data are provided as a Source Data file.

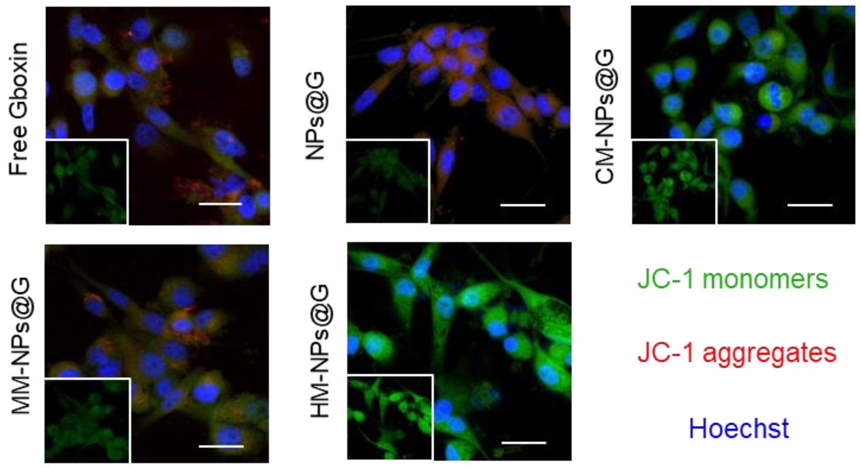

Supplementary Figure 13 Mitochondria membrane potential assay after HM-NPs@G treatment by JC-1 kit. CLSM images of U87MG cells stained with JC-1 after treatment with different formulations for 72 h. (JC-1 monomers (green), JC-1 aggregates (red), Hoechst (blue)). Scale bar = 20 μm. The potential analyses were representative data from three independent experiments.

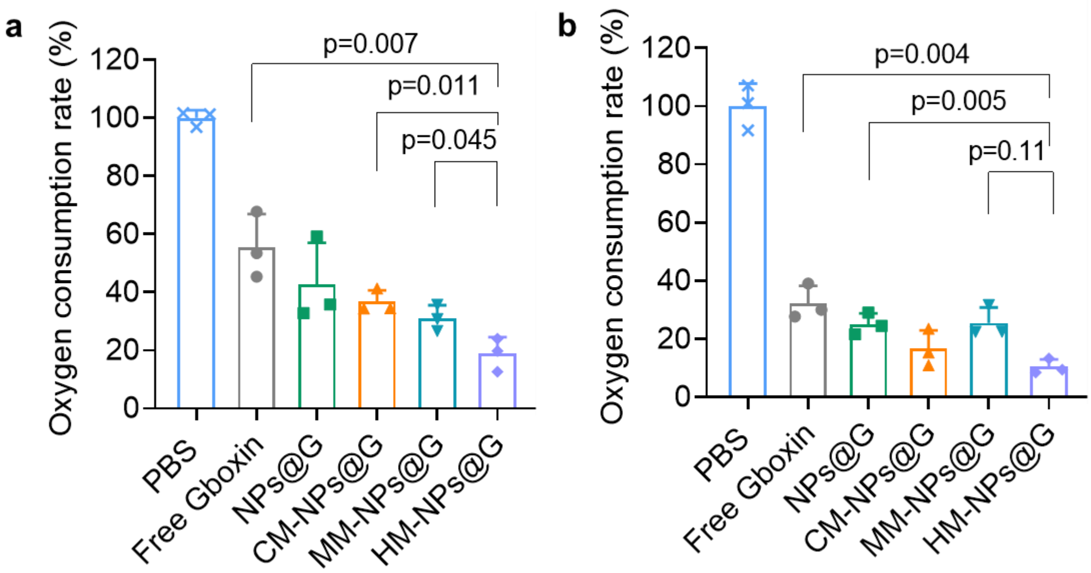

Supplementary Figure 14 Oxygen consumption rate analysis in U87MG and GSCs X01 cells in vitro. The rate of oxygen consumption within 2 h in U87MG (n = 3 biologically independent samples) **(a)** and X01 cells (n = 3 biologically independent samples) **(b)** treated with HM-NPs@G, MM-NPs@G, CM-NPs@G, NPs@G and free Gboxin was analysed. Data are presented as mean ± SD (one-way ANOVA and Tukey multiple comparisons tests). Source data are provided as a Source Data file.

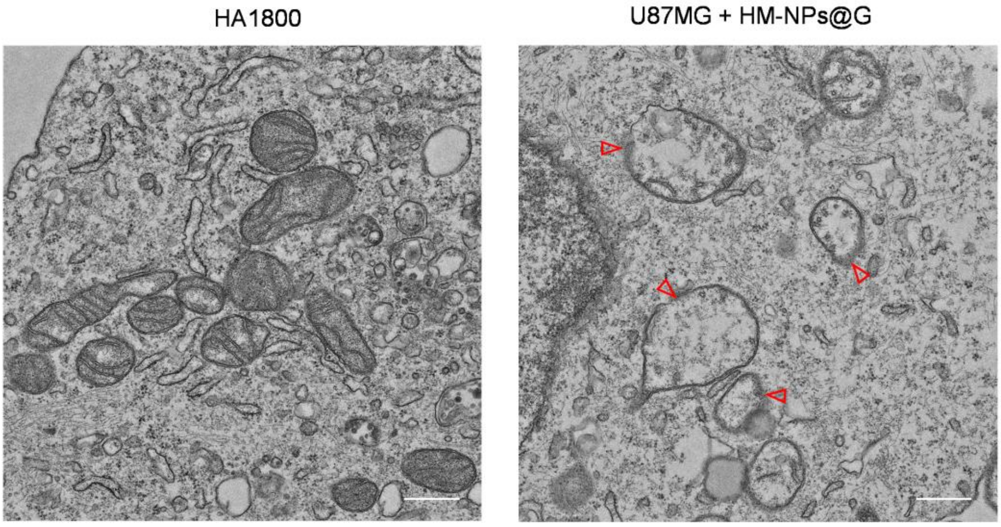

Supplementary Figure 15 Mitochondria morphology analysis of normal cells and HM-NPs@G treated GBM cells using Bio-TEM. TEM images of mitochondria in normal cells (HA1800) and U87MG cells treated with HM-NPs@G. The red arrows indicated the damaged construction of mitochondria after treatment of nanomedicines. Scale bar = 500 nm. The TEM images were representative data from three independent experiments.

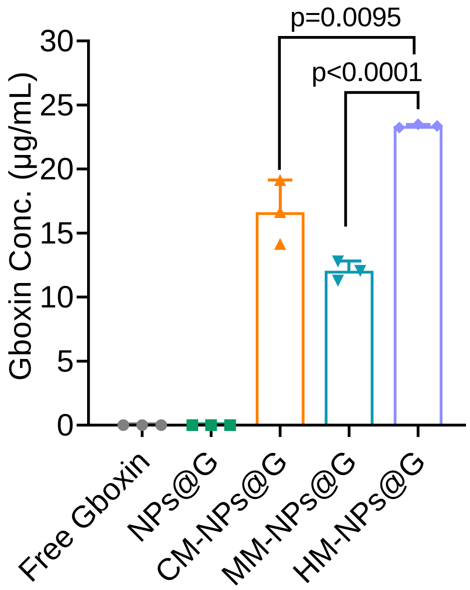

Supplementary Figure 16 Gboxin accumulation ratio analysis in the mitochondria of U87MG cells treated with different formulations in vitro. The accumulation ratio of Gboxin was detected with HPLC in mitochondria of U87MG cells treated with HM-NPs@G, MM-NPs@G, CM-NPs@G, NPs@G and free Gboxin for 24 h (n=3 biologically independent samples). Data are presented as mean ± SD (one-way ANOVA and Tukey multiple comparisons tests). Source data are provided as a Source Data file.

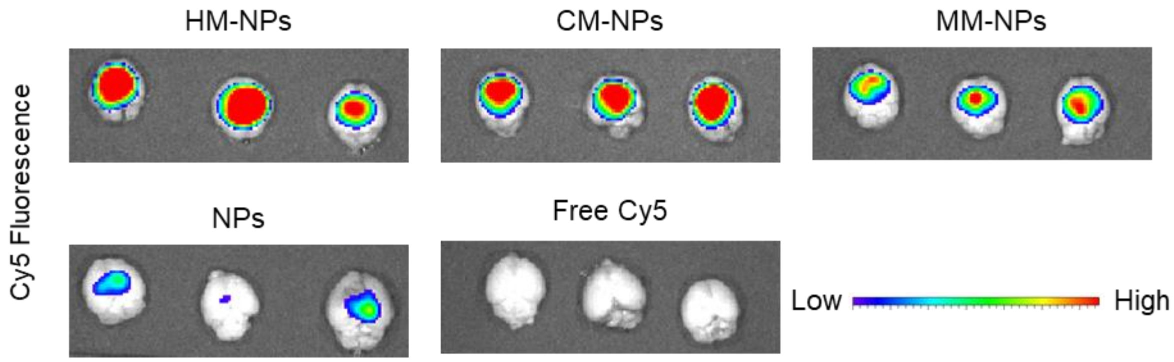

Supplementary Figure 17 Ex vivo imaging analysis. Cy5 fluorescence images of GBM brains taken from U87MG-Luc bearing mice at 6 h post i.v. injection of HM-NP@Cy5 (1 mg Cy5 equiv. kg^{-1}, n = 3 mice in each group).

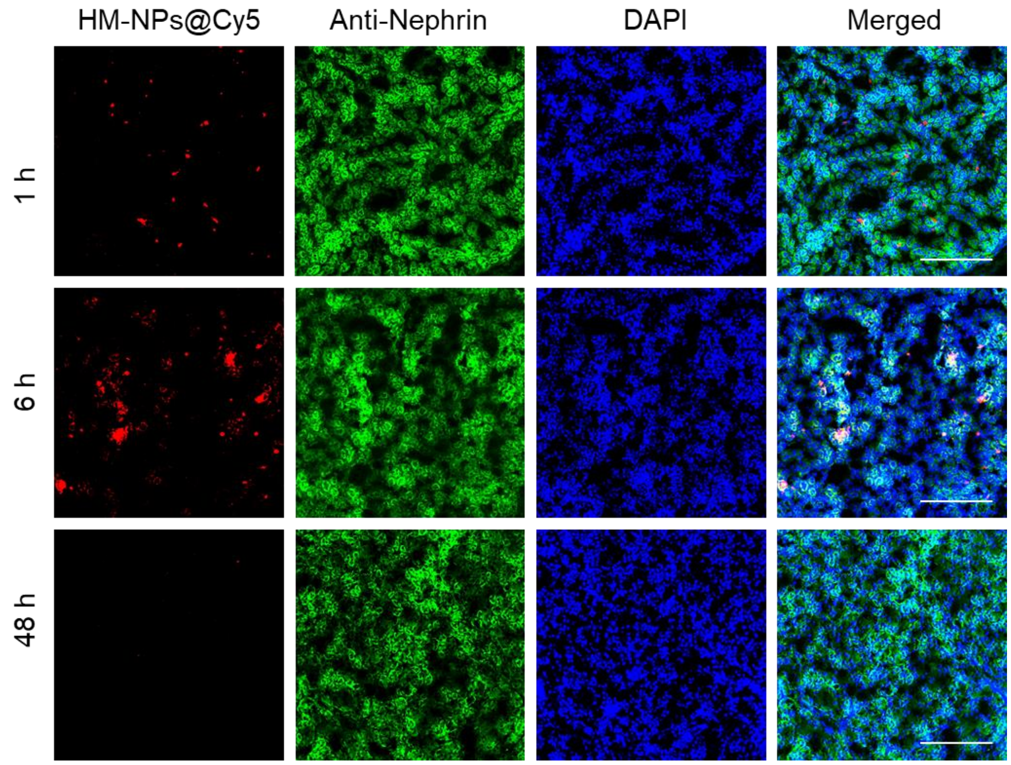

Supplementary Figure 18 Tissue-level distribution in renal corpuscles of HM-NPs. Immunofluorescence images of kidney taken from healthy BALB/c mice 1 h, 6 h, and 48 h post injection of HM-NPs@Cy5 (2 mg Cy5 equiv. kg^{-1}). (Nuclei were stained with DAPI (blue) and renal glomerulus with Anti-Nephrin Rabbit pAb (green); Cy5 fluorescence is red. Scale bars = 100 μm. The immunofluorescence analyses were representative data from three mice.

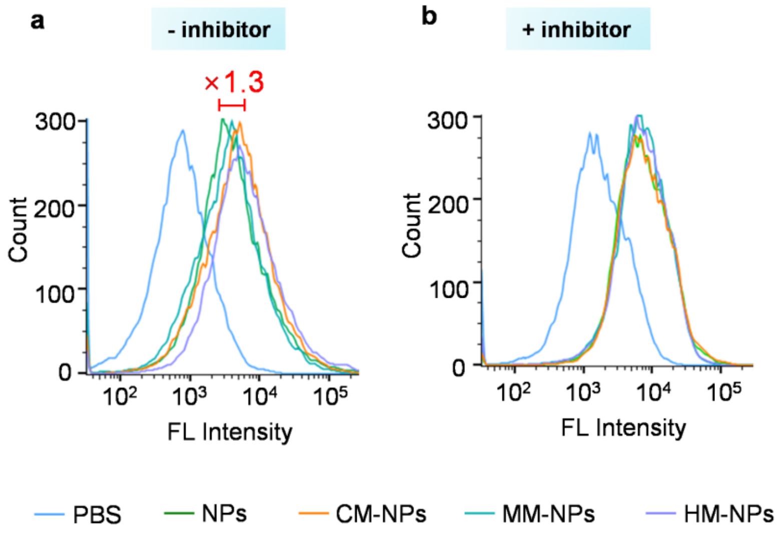

Supplementary Figure 19 BBB penetration and cellular uptake analysis in vitro. The fluorescence intensities were detected with flow cytometry of U87MG cells after nanoparticles traversed the BBB without **(a)** or with **(b)** 8-CPT-cAMP and Ro 20-1724 pretreatments (FITC concentration: 10 μg mL^{-1}). The flow cytometry analyses were representative data from three independent experiments.

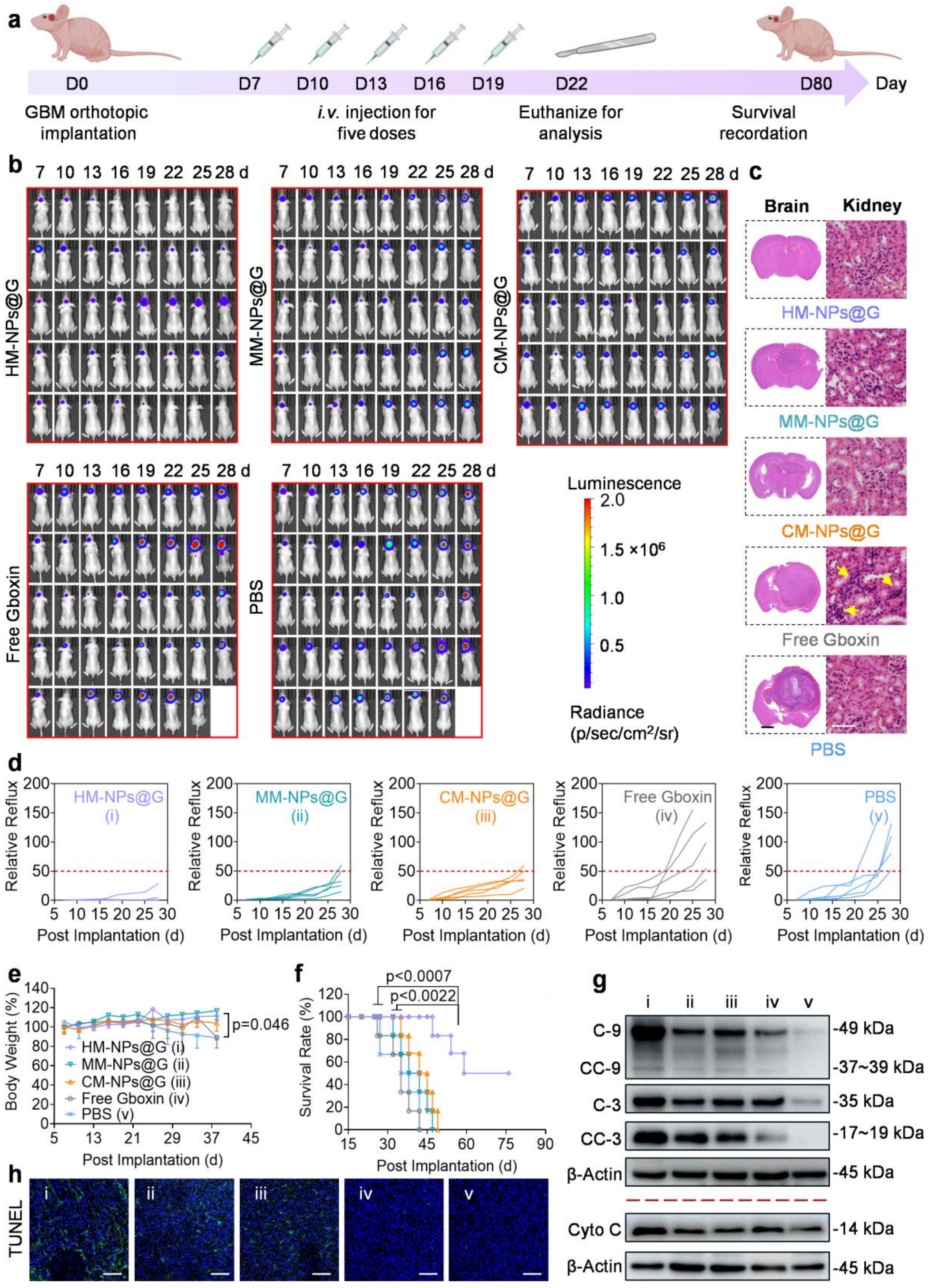

Supplementary Figure 20 In vivo antitumour activity of HM-NPs@G in mice bearing orthotopic U87MG-Luc GBM tumour. a, Schematic illustration shows study treatment

schedule. **b**, Time course bioluminescence images of mice bearing orthotopic U87MG-Luc bearing tumour and treated with HM-NPs@G, MM-NPs@G, CM-NPs@G, free Gboxin, or PBS (n = 5 mice in each group). The mice were intravenously injected at a dose of 3 mg Gboxin equiv. kg^{-1} on days 7, 10, 13, 16, and 19 post tumour implantations. **c**, H&E images of the whole brain (Scale bar = 2 mm) and kidney (Scale bar = 40 μm) excised from the representative mice in following treatments as described above on day 22. The histological analyses were representative data from three mice. **d**, Quantified luminescence levels of mice using the Lumina IVIS III system (n = 5 mice in each group). **e**, Body weight profile assessment p (n = 5 mice in each group). Data are presented as mean ± SD (one-way ANOVA and Tukey multiple comparisons tests). **f**, Mice survival rate curves. Statistical analysis: HM-NPs@G vs. MM-NPs@G or CM-NPs@G, *$p < 0.05$, HM-NPs@G vs. free Gboxin or PBS, **$p < 0.01$ (n=6 mice in each group, Kaplan-Meier analysis, log-rank test). **g,** Western blot analysis of apoptosis associated proteins and Cyto C in tumour tissues excised from the mice on day 22 The immunoblots were representative data from three independent experiments. **h**, Histological analysis using the TUNEL assay The histological analyses were representative data from three mice. Green: apoptotic cells; blue: Hoechst-stained cell nuclei (scale bar = 100 μm). Source data are provided as a Source Data file.

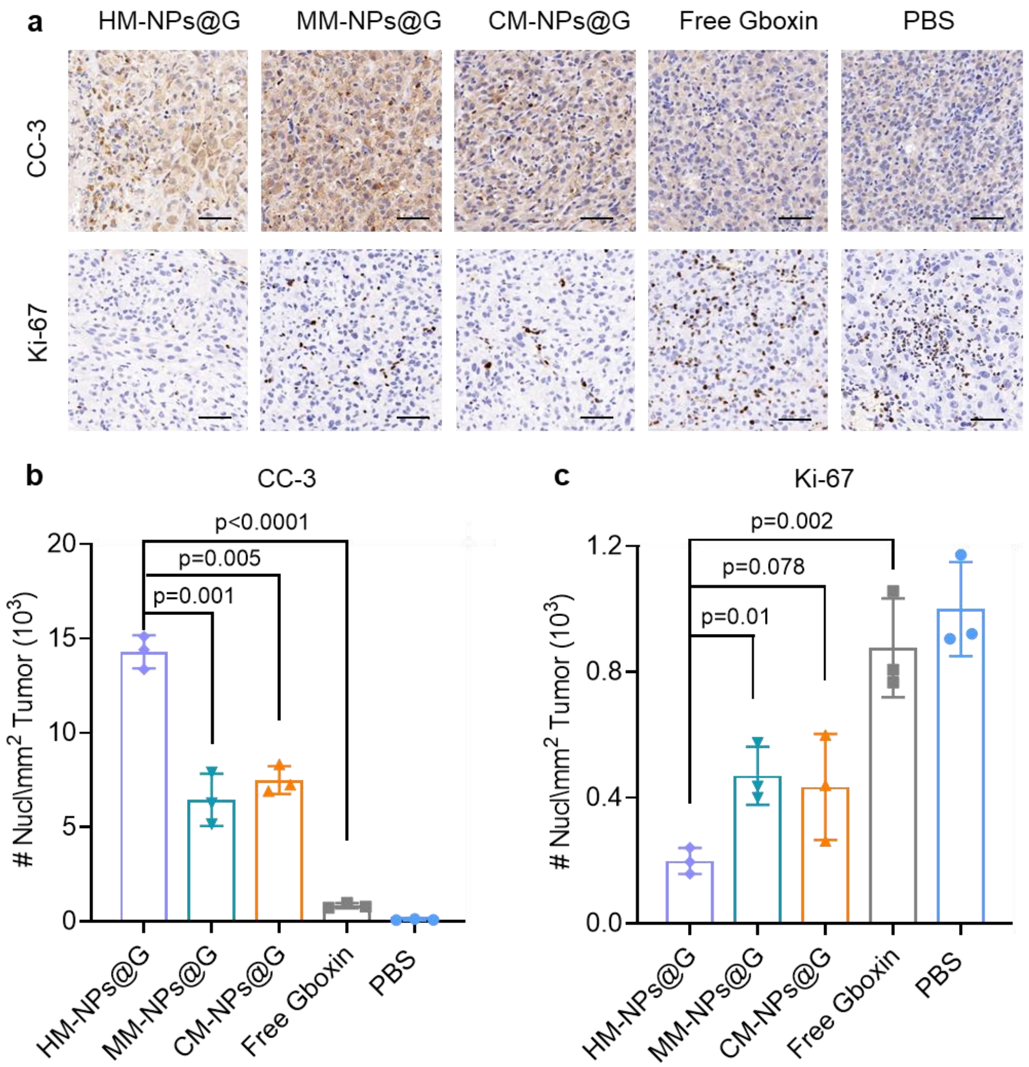

Supplementary Figure 21 Apoptosis and proliferation immunohistochemistry analysis of tumors after different treatments. (a) Cleaved caspase 3 (CC-3) and proliferation (Ki-67) immunohistochemistry of tumor slices excised from U87MG-Luc mice following different treatments. Quantification of tumor cells staining positive for CC-3 **(b)** and Ki-67 **(c)**, Signal intensity was quantified from > 300 cells in tumors from three mice per treatment condition using ImageJ (n =3). Data are presented as mean ± SD (one-way ANOVA and Tukey multiple comparisons tests). Scale bar = 50 μm. The immunohistochemistry analyses were representative data from three mice. Source data are provided as a Source Data file.

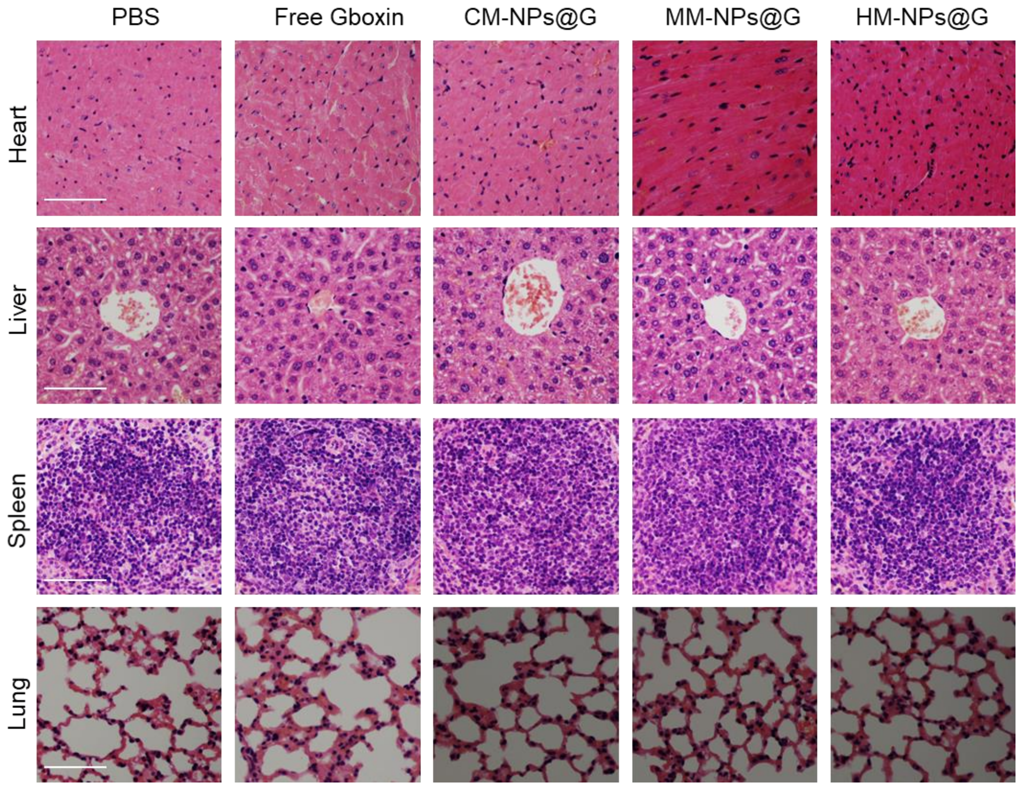

Supplementary Figure 22 Histological analysis of major organs from mice treated with different treatments. Histological analyses of the major organs excised from orthotopic U87MG-Luc human glioblastoma tumor-bearing nude mice following different treatments. Images were obtained using an Olympus microscope using a 40 × objective (Scale bar = 40 μm). The histological analyses were representative data from three mice.

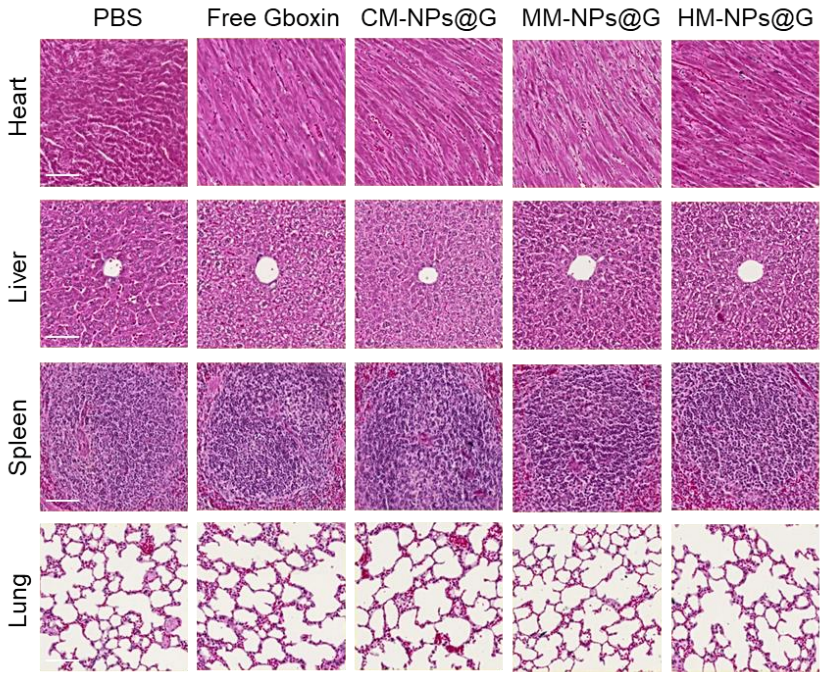

Supplementary Figure 23 Histological analysis after treatments with different formulations. Histological analyses of the major organs excised from orthotopic X01-Luc human GSCs bearing nude mice following different treatments. Images were obtained using an Olympus microscope using a 40 × objective (scale bar = 40 μm). The histological analyses were representative data from three mice.

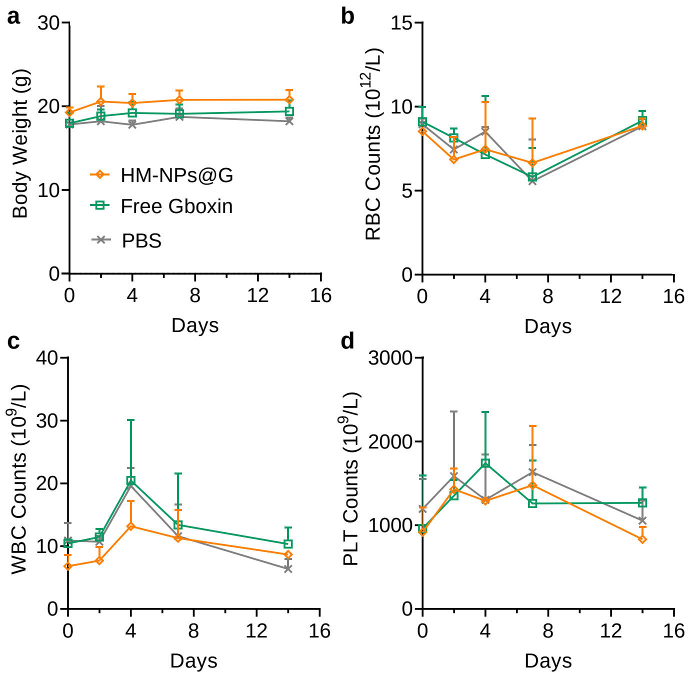

Supplementary Figure 24 Safety evaluation of HM-NPs@G in vivo. (a) Body weight changes and blood parameters analysis (**(b)** RBC counts, **(c)** WBC counts, and **(d)** PLT counts) of BALB/c healthy mice treated with HM-NPs@G, free Gboxin (5 mg Gboxin equiv. kg^{-1}) or PBS at day 0, 2, 4, 7 or 14 (n = 3 mice in each group). Data are presented as mean ± SD (one-way ANOVA and Tukey multiple comparisons tests). Source data are provided as a Source Data file.

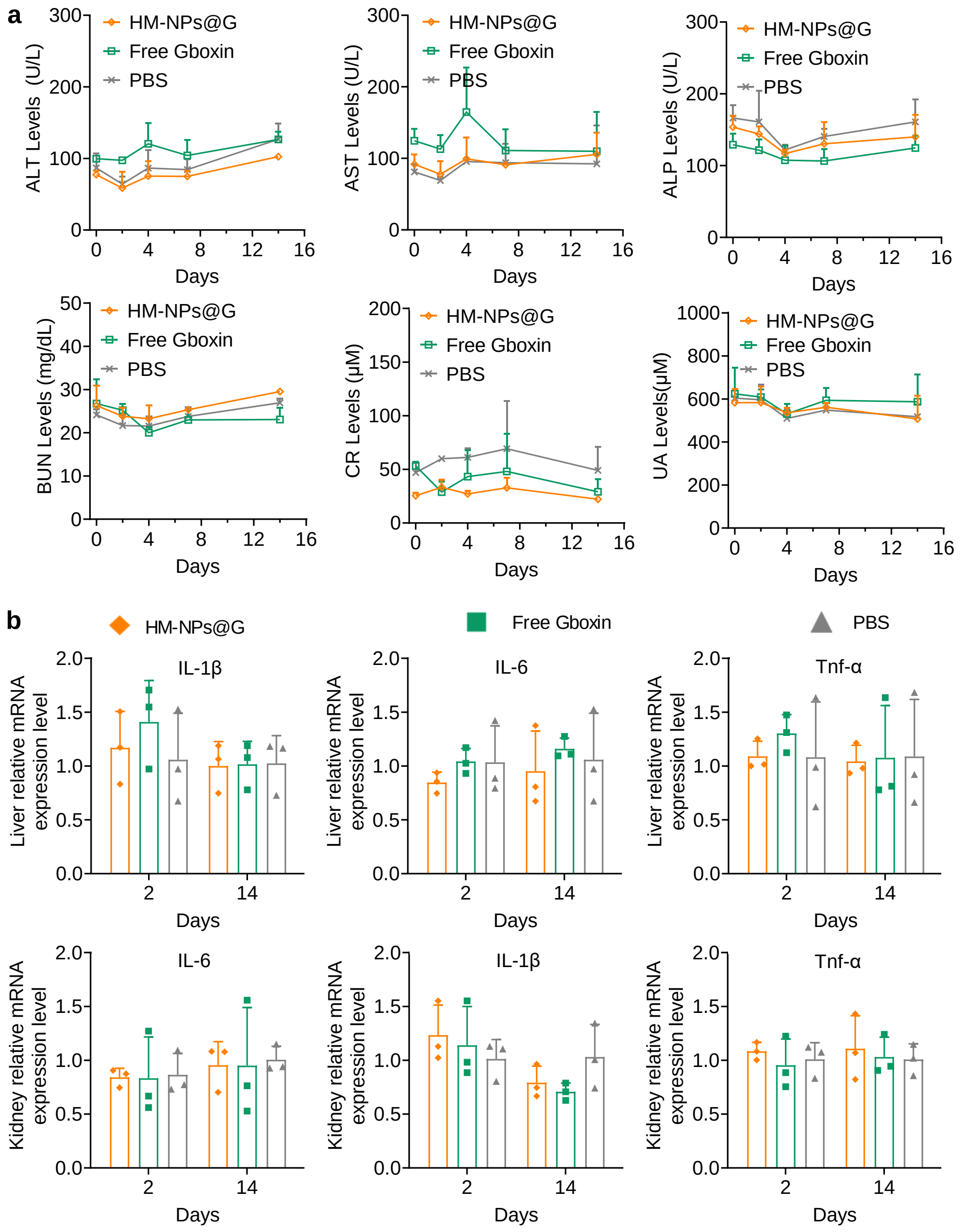

Supplementary Figure 25 Safety evaluation of HM-NPs@G in vivo. (a) Blood biochemistry examinations. Assessment of mouse plasma alanine aminotransferase (ALT), aspartate aminotransferase (AST), alkaline phosphatase (ALP), plasma urea (BUN), creatinine (CR), and uric acid (UA) levels after a single-dose nanomedicine treatment. **(b)** Core proinflammatory cytokines such as IL-1β, IL-6, and Tnf-α in the liver and kidney were assessed after being treated with HM-NPs@G, free Gboxin, or PBS at day 2, or day 14 (n = 3

mice in each group). Data are presented as mean ± SD (one-way ANOVA and Tukey multiple comparisons tests). Source data are provided as a Source Data file.

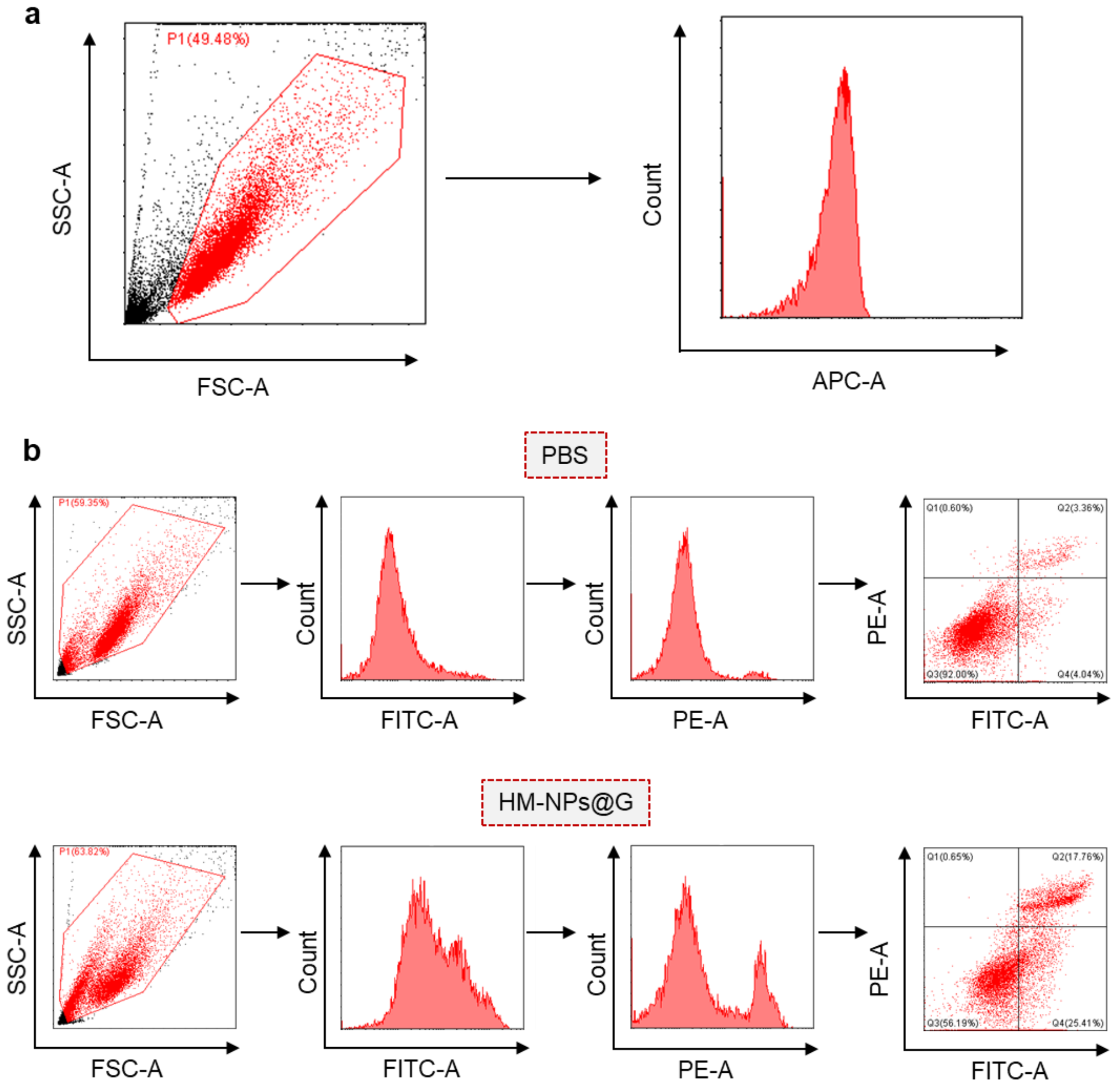

Supplementary Figure 26 Gating strategy of flow cytometry. The gating strategy of flow cytometry analysis for the **(a)** cell uptake and **(b)** apoptosis in U87MG cells.

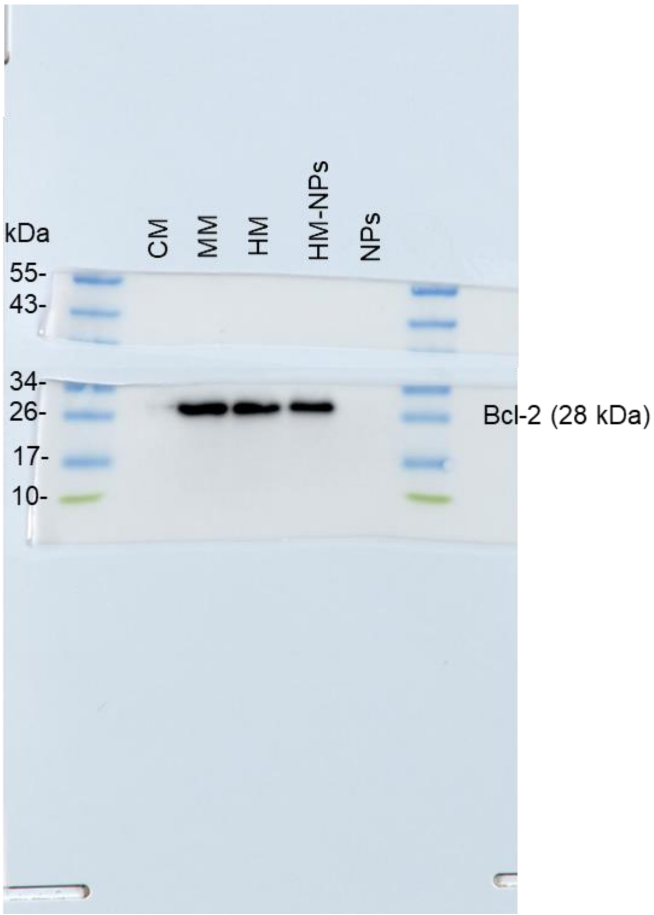

Supplementary Figure 27 Uncropped scans of blots. Pieced together scan of the Supplementary Figure 5.
