# Supplementary material for: Cancer cell-mitochondria hybrid membrane coated Gboxin loaded nanomedicines for glioblastoma treatment
Source: Nat Commun. 2023 Jul 28;14:4557. doi: 10.1038/s41467-023-40280-3 (PMC10382535; doi:10.1038/s41467-023-40280-3)
Supplement: Supplementary file 3 — Reporting Summary [file 41467_2023_40280_MOESM3_ESM.pdf]

nature portfolio

Corresponding author(s): Bingyang Shi

Last updated by author(s): Jul 10, 2023

Reporting Summary

Nature Portfolio wishes to improve the reproducibility of the work that we publish. This form provides structure for consistency and transparency in reporting. For further information on Nature Portfolio policies, see our Editorial Policies and the Editorial Policy Checklist.

Statistics

For all statistical analyses, confirm that the following items are present in the figure legend, table legend, main text, or Methods section.

n/a	Confirmed	
☐	☒	The exact sample size (n) for each experimental group/condition, given as a discrete number and unit of measurement
☐	☒	A statement on whether measurements were taken from distinct samples or whether the same sample was measured repeatedly
☐	☒	The statistical test(s) used AND whether they are one- or two-sided<br>*Only common tests should be described solely by name; describe more complex techniques in the Methods section.*
☒	☐	A description of all covariates tested
☒	☐	A description of any assumptions or corrections, such as tests of normality and adjustment for multiple comparisons
☐	☒	A full description of the statistical parameters including central tendency (e.g. means) or other basic estimates (e.g. regression coefficient) AND variation (e.g. standard deviation) or associated estimates of uncertainty (e.g. confidence intervals)
☐	☒	For null hypothesis testing, the test statistic (e.g. F, t, r) with confidence intervals, effect sizes, degrees of freedom and P value noted<br>*Give P values as exact values whenever suitable.*
☒	☐	For Bayesian analysis, information on the choice of priors and Markov chain Monte Carlo settings
☒	☐	For hierarchical and complex designs, identification of the appropriate level for tests and full reporting of outcomes
☒	☐	Estimates of effect sizes (e.g. Cohen's d, Pearson's r), indicating how they were calculated

Our web collection on statistics for biologists contains articles on many of the points above.

Software and code

Policy information about availability of computer code

Data collection	Data were collected using AVANCE III HD 400MHz, Malvern Zetasizer Nano ZS, JEOL JEM-F200, Zeiss 880, Beckman (CytoFLEX), Devices/13x, GE Amersham Imager 680RGB, Olympus FV3000, Agilent 1260Infinity II prime, IVIS Lumia III.
Data analysis	Data analysis was conducted using Microsoft Excel (2016), GraphPad Prism (Version 8), Origin 2021, MestReNov 5.3.1-4696, Zetasizer Software v7.11, Living Image 4.5.5, ZEN 2.3 SP1, ImageJ 1.37v , CytExpert 2.3, QuPath 0.1.2.

For manuscripts utilizing custom algorithms or software that are central to the research but not yet described in published literature, software must be made available to editors and reviewers. We strongly encourage code deposition in a community repository (e.g. GitHub). See the Nature Portfolio guidelines for submitting code & software for further information.

Data

Policy information about availability of data

All manuscripts must include a data availability statement. This statement should provide the following information, where applicable:

- Accession codes, unique identifiers, or web links for publicly available datasets
- A description of any restrictions on data availability
- For clinical datasets or third party data, please ensure that the statement adheres to our policy

All data generated or analyzed during this study are available within the Article and its Supplementary Information file and the Source data file. Source data are provided with this paper.

Research involving human participants, their data, or biological material

Policy information about studies with human participants or human data. See also policy information about sex, gender (identity/presentation), and sexual orientation and race, ethnicity and racism.

Reporting on sex and gender	Our article does not include any human participants or human data.
Reporting on race, ethnicity, or other socially relevant groupings	Our article does not include any human participants or human data.
Population characteristics	Our article does not include any human participants or human data.
Recruitment	Our article does not include any human participants or human data.
Ethics oversight	Our article does not include any human participants or human data.

Note that full information on the approval of the study protocol must also be provided in the manuscript.

Field-specific reporting

Please select the one below that is the best fit for your research. If you are not sure, read the appropriate sections before making your selection.

[x] Life sciences [ ] Behavioural & social sciences [ ] Ecological, evolutionary & environmental sciences

For a reference copy of the document with all sections, see nature.com/documents/nr-reporting-summary-flat.pdf

Life sciences study design

All studies must disclose on these points even when the disclosure is negative.

Sample size	Details regarding the sample size of all experiments are provided in figure legends. Sample size was estimated to achieve about 90% power for detection of significant differences in tumor volume between groups based on means and standard deviations in preliminary studies. All available samples passing the quality control were included.
Data exclusions	No data were excluded from analysis.
Replication	Experiments were repeated at least three times with similar results. All attempts at replication were successful.
Randomization	In vitro cell experiments were seeded identically at the onset of the experiments and randomized into the various treatment groups prior to the beginning of treatment protocols.<br><br>For in vivo studies, once mice were implanted with tumor tissues/cells they were randomized into experimental groups of 3-9 mice prior to treatment administration.
Blinding	The investigators and authors have been consistently blinded to the group allocation during data collection and analysis.

Reporting for specific materials, systems and methods

We require information from authors about some types of materials, experimental systems and methods used in many studies. Here, indicate whether each material, system or method listed is relevant to your study. If you are not sure if a list item applies to your research, read the appropriate section before selecting a response.

Materials & experimental systems

n/a	Involved in the study	
[ ]	[x]	Antibodies
[ ]	[x]	Eukaryotic cell lines
[x]	[ ]	Palaeontology and archaeology
[ ]	[x]	Animals and other organisms
[x]	[ ]	Clinical data
[x]	[ ]	Dual use research of concern
[x]	[ ]	Plants

Methods

n/a	Involved in the study	
[x]	[ ]	ChIP-seq
[ ]	[x]	Flow cytometry
[x]	[ ]	MRI-based neuroimaging

Antibodies

Antibodies used	Western blotting: Anti-Cytochrome C rabbit antibody (Abcam, Catalog no.ab133504, 1/5000 dilution), Anti-Claudin 5 rabbit antibody (Abcam, Catalog no.ab131259, 1/5000 dilution), Caspase-9 Antibody (Human Specific) rabbit antibody (cell signaling technology, Catalog no.9502S, 1/1000 dilution), Caspase-3 rabbit antibody (cell signaling technology, Catalog no.9662S, 1/1000 dilution) and Cleaved Caspase-3 (Asp175) (5A1E) rabbit antibody (cell signaling technology, Catalog no.9664S, 1/1000 dilution), ZO-1 rabbit Polyclonal antibody (Beyotime, Catalog no.AF8394, 1/1000 dilution), Anti-CD44 rabbit antibody (Abcam, Catalog no. ab243894, 1/1000 dilution), Anti-EpCAM rabbit antibody (Abcam, Catalog no. ab223582, 1/1000 dilution), Anti-EHD2 rabbit antibody (Abcam, Catalog no. ab154784, 1/5000 dilution), Anti-Mitofusin rabbit antibody (Abcam, Catalog no. ab221661, 1/1000 dilution), Atlastin-1 rabbit antibody (cell signaling technology, Catalog no. 12728S, 1/1000 dilution), Integrin αV rabbit antibody (cell signaling technology, Catalog no. 60896S, 1/1000 dilution), Na,K-ATPase rabbit antibody (cell signaling technology, Catalog no. 3010S, 1/1000 dilution), β-actin rabbit antibody (Thermofisher, Catalog no.BS-50545R, 1/5000 dilution).<br><br>Immunohistochemistry staining: Ki67 rabbit polycional antibody (Servicebio, Catalog no.GB111499, 1:500 dilution), Cleaved Caspase 3 rabbit polyclonal antibody (Servicebio, Catalog no.GB11532, 1:500 dilution).<br><br>Immunofluorescence analysis: Anti-hsp60 rabbit polyclonal antibody (Servicebio, Catalog no.GB11243, 1/1000 dilution), Anti-Nephrin rabbit polyclonal antibody (Servicebio, Catalog no. GB11343, 1/1000 dilution).<br><br>Secondary antibodies: HRP Goat Anti-Rabbit IgG (H&L) (UElandy, Catalog no. H6162S/H6162, 1/25000 dilution), HRP Goat Anti-Mouse IgG (H&L) (UElandy, Catalog no. H6161S/H6161, 1/25000 dilution).
Validation	Antibodies used were commercially available and were validated in multiple previous studies. The validation of therapeutic antibodies were performed by SDS-PAGE or Bio X Cell, with relevant data presented on the manufacturer's website. The following is website of all above antibodies.<br>https://www.abcam.cn/products/primary-antibodies/cytochrome-c-antibody-epr1327-ab133504.html<br>https://www.abcam.cn/products/primary-antibodies/claudin-5-antibody-epr7583-ab131259.html<br>https://www.cellsignal.com/products/primary-antibodies/caspase-9-antibody-human-specific/9502<br>https://www.cellsignal.com/products/primary-antibodies/caspase-3-antibody/9662<br>https://www.cellsignal.com/products/primary-antibodies/cleaved-caspase-3-asp175-5a1e-rabbit-mab/9664<br>https://www.beyotime.com/product/AF8394.htm<br>https://www.abcam.cn/products/primary-antibodies/cd44-antibody-blr038f-ab243894.html<br>https://www.abcam.cn/products/primary-antibodies/epcam-antibody-epr20532-225-ab223582.html<br>https://www.abcam.cn/products/primary-antibodies/ehd2-antibody-ab23935.html<br>https://www.abcam.cn/products/primary-antibodies/mitofusin-1-antibody-epr21953-74-ab221661.html<br>https://www.cellsignal.cn/products/primary-antibodies/atlastin-1-d2e6-rabbit-mab/12728<br>https://www.cellsignal.com/products/primary-antibodies/integrin-av-d2n5h-rabbit-mab/60896<br>https://www.cellsignal.com/products/primary-antibodies/na-k-atpase-antibody/3010<br>https://www.thermofisher.com/antibody/product/beta-actin-Antibody-Polyclonal/BS-50545R

Eukaryotic cell lines

Policy information about cell lines and Sex and Gender in Research

Cell line source(s)	The hCMEC/D3 cell line, HA1800 cell line, N2a cell line, BV2 cell line, U251 cell line, U251TR cell line and U87MG cell line were purchased from the American Type Culture Collection (ATCC). U87MG-Luc cell line was purchased from Shanghai Model Organisms Center, Inc. The X01 cell line was provided by our colleague Prof. Jinlong Yin.
Authentication	The cell lines were morphologically confirmed according to the information provided by ATCC and Shanghai Model Organisms Center, Inc. The X01 cells were authenticated by short tandem repeat (STR) analysis.
Mycoplasma contamination	All the cell lines used in this study were mycoplasma contamination free.
Commonly misidentified lines<br>(See ICLAC register)	No commonly misidentified cell lines were used in this study.

Animals and other research organisms

Policy information about studies involving animals; ARRIVE guidelines recommended for reporting animal research, and Sex and Gender in Research

Laboratory animals	Laboratory animals are BALB/c female nude mice (6-8 weeks, 18-20 g) that were purchased from SPF (Beijing) Biotechnology Co., Ltd. Mouse room has a 12 h light-dark cycle. The room temperature is between 20-24℃. The relative humidity is kept at 45-65%.
Wild animals	No wild animals were used in this research.
Reporting on sex	Female
Field-collected samples	No field-collected samples were used in this research.

Ethics oversight	All animal handling protocols and experiments were approved by the Medical and Scientific Research Ethics Committee of Henan University School of Medicine (P. R. China) (HUSOM-2018-355).

Note that full information on the approval of the study protocol must also be provided in the manuscript.

Flow Cytometry

Plots

Confirm that:

- [x] The axis labels state the marker and fluorochrome used (e.g. CD4-FITC).
- [x] The axis scales are clearly visible. Include numbers along axes only for bottom left plot of group (a 'group' is an analysis of identical markers).
- [x] All plots are contour plots with outliers or pseudocolor plots.
- [x] A numerical value for number of cells or percentage (with statistics) is provided.

Methodology

Sample preparation	U87MG cells were seeded in 12-well plates (5 × 105 cells/well). After incubation for 24 h, the cells were treated with Cy5 loaded HM-NPs, MM-NPs, CM-NPs, NPs (Cy5: 10 μg mL-1) and incubated for 6 h. PBS-treated cells were used as control. The cells were washed three times with cold PBS, harvested and recorded immediately using a flow cytometer (Beckman, CytoFLEX), then analyzed using Cell Quest software based on 10,000 gated events. The gate was arbitrarily set for the detection of Cy5 fluorescence.<br><br>U87MG or X01 cells (1 × 105 cells/well) were seeded in the 12-well plates for 24 h and cultured with HM-NPs@G, CM-NPs@G, MM-NPs@G, NPs@G and free Gboxin (Gboxin: 800 nM). After 72 h incubation, the U87MG or X01 cells were washed 3 times with PBS, and stained with 10 μL iodide and 5 μL Annexin V-FITC, cultured for 15 min in the dark. The cells were resuspended in 300 μL PBS followed by analyzing with a flow cytometer (Beckman, CytoFLEX).
Instrument	Beckman (CytoFLEX)
Software	CytExpert v2.3 software
Cell population abundance	No sorting was performed.
Gating strategy	Gating strategy are referred to those described in the Beckman website. Gating was based on FSC/SCC together with viability dyes and singlet populations. The cell populations within the gate were further analysed based on the expression of markers.

- [ ] Tick this box to confirm that a figure exemplifying the gating strategy is provided in the Supplementary Information.
